# Perioperative Myocardial Infarction Following Dabigatran Reversal With Idarucizumab in a Patient Undergoing Orthotopic Liver Transplantation

**DOI:** 10.7759/cureus.43531

**Published:** 2023-08-15

**Authors:** Alex Barroso, M. Carmen Martinez-Gonzalez, Nathanael Knowlson, Alba M. Miguel, Gonzalo Perez

**Affiliations:** 1 Anesthesiology and Reanimation, Hospital Regional Universitario de Málaga, Málaga, ESP; 2 Intensive Care Unit, Hospital Regional Universitario de Málaga, Málaga, ESP

**Keywords:** orthotopic liver transplantation, dabigatran, myocardial infarction, anaesthesia, viscoelastic test, idarucizumab, perioperative myocardial infarction, liver transplantation

## Abstract

Insufficient information is available regarding the administration of anticoagulants, specifically direct oral anticoagulants, in individuals with cirrhosis awaiting liver transplantation. In this report, we present a case of a 66-year-old male with atrial fibrillation treated with dabigatran who received idarucizumab prior to orthotopic liver transplantation. Hemostatic status was monitored throughout the procedure with both conventional hemostatic tests and point-of-care viscoelastic hemostatic assays. The patient suffered an intraoperative myocardial infarction, which could be related to the use of idarucizumab.

## Introduction

Direct oral anticoagulants (DOACs) are increasingly used to treat and prevent thromboembolism in patients with end-stage liver disease (ESLD), and despite this fact, this group is generally excluded from the larger trials. Evidence of safety and efficacy is slowly emerging for this population. Dabigatran is a factor-IIa inhibitor approved for the treatment of venous thromboembolism and to decrease the risk of stroke and systemic embolism in patients with non-valvular atrial fibrillation [1]. This drug has a direct reversal agent available, i.e., idarucizumab. There are rare reports showing significant prothrombotic side effects after using idarucizumab, such as venous thrombosis, strokes, or even myocardial infarction [2].

The assessment of the patient's hemostatic status during orthotopic liver transplantation (OLT) has evolved since viscoelastic hemostatic assays (VHA) have become widely available. VHA are point-of-care tests that allow a global assessment of coagulation using whole blood. The technology enabling this assessment has evolved from the original thromboelastography to the most recent Quantra Hemostasis Analyzer (HemoSonics, LLC, Durham, NC) [3,4]. Several groups have assessed the utility of Quantra when compared to other VHA and standard laboratory tests in adult patients undergoing cardiac surgery and trauma patients. However, there is scarce literature on the setting of liver transplantation and in particular its role in monitoring the reversal of dabigatran with idarucizumab. The patient provided written consent for every action clinically taken and for the publication of this case report.

## Case presentation

**Figure 1:**
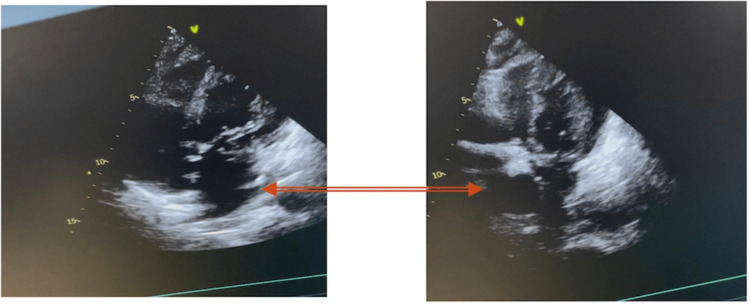
Presurgical echocardiography Images in systole and diastole show normal ejection fraction (55%), with no regional wall abnormalities or valvular defects.

We present a 66-year-old male with alcohol and hepatitis C virus (HCV)-related ESLD, with a Model for End-Stage Liver Disease (MELD) score of 14. He had previously undergone a segment VI laparoscopic liver resection as treatment for a hepatic tumor without perioperative complications. The patient also presented mild chronic obstructive pulmonary disease and emphysema, hypertension, diabetes mellitus type 2, dyslipidemia, and stable paroxysmal atrial fibrillation. He also received oral anticoagulation with dabigatran 150 mg per day. The patient had no previous major cardiovascular events and a negative history of chest pain. Cardiovascular risk factors such as hypertension and diabetes were being treated by a primary care physician and were considered under control with medication (metformin and enalapril). The patient also received propranolol as primary prevention for esophageal varices bleeding. A transthoracic echocardiogram (TTE) performed as part of the preoperative protocol showed a normal ejection fraction (55%), with no regional wall abnormalities or valvular defects (Figure 1). A functional stress test was also performed with no abnormal findings.

When a compatible organ was selected for transplantation, he did not have time to discontinue dabigatran, taking his last dose approximately nine hours before surgery. The patient was successfully put under general anesthesia, and the process of intubation was carried out without any complications. Before the insertion of arterial and central venous catheters, a solitary intravenous dose of 5 grams of idarucizumab was administered.

**Figure 2:**
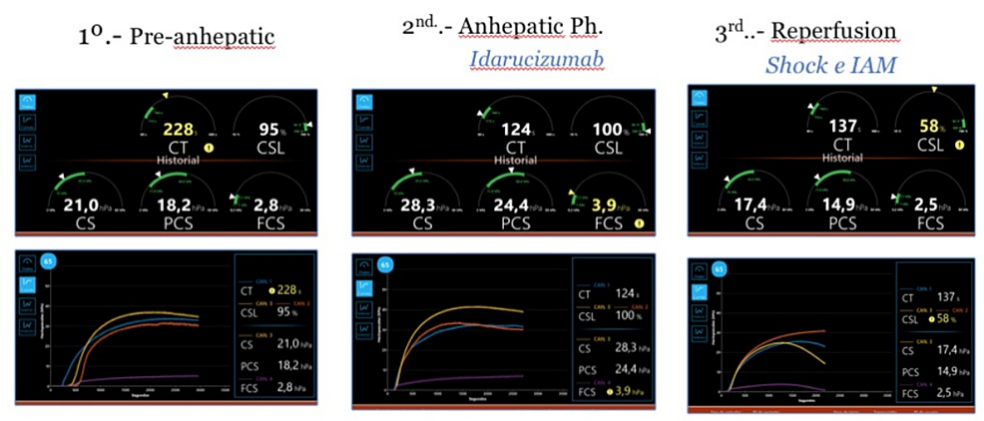
Viscoelastic hemostatic assay The image shows the Quantra® System, which is based on SEER (Sonic Estimation of Elasticity via Resonance) sonorheometry. Images show graphically the information shared in Table 1. The left images represent the study before the surgical incision. Images at the center show the mechanical properties of the clot after the use of idarucizumab. The right images aim to show the hemostasis status after the reperfusion phase. CT: clot time; CSL: clot stability to lysis; CS: clot stiffness; PCS: platelet contribution to clot stiffness; FCS: fibrinogen contribution to clot stiffness.

Before using idarucizumab, the usual tests were performed, showing hemoglobin (Hgb) of 11.3 g/dL and a platelet count of 121.000 cells/mm^3^, as well as a viscoelastic control on a Quantra Analyzer. VHA allowed us to verify the optimal reversal of dabigatran after 50 minutes of idarucizumab infusion. The coagulation status was also validated clinically with the surgeons, as no abnormal bleeding was observed in the surgical field (Tables 1, 2 and Figure 2).

**Table 1:** Hemostasis analysis, Quantra parameters QStat parameters: clot time (CT), clot stiffness parameters (CS, FCS, and PCS), and clot stability to lysis (CSL). VHA: viscoelastic hemostatic assay; CS: clot stiffness; PCS: platelet contribution to clot stiffness; FCS: fibrinogen contribution to clot stiffness.

VHA Quantra parameters (references)	Pre-idarucizumab	Post-idarucizumab (50 minutes)	After portal reperfusion
CT (113-164 s)	228 s	124 s	137 s
CSL (95%-100%)	95%	100%	58%
CS (13-33.2 hPa)	21 hPa	28.3 hPa	17.4 hPa
PCS (11.9-29.8 hPa)	18.2 hPa	24.4 hPa	15.1 hPa
FCS (1-3.7 hPa)	2.8 hPa	3.7 hPa	2.5 hPa

**Table 2:** Hemostasis analysis, blood parameters INR: international normalized ratio; PT: prothrombin time; aPTT: activated partial thromboplastin time; TT: thrombin time.

Hemostatic parameter	Pre-idarucizumab	Post-idarucizumab (50 minutes)	After portal reperfusion	Postsurgical/ICU
INR (0.9-1.1)	1.6	1.18	1.3	1.5
PT (11-15 sec.)	18.3	14.1	16.1	19.1
aPTT (20-31 sec.)	46.5	25.8	41.6	45.2
TT (14-21 sec.)	120	19.5	27.7	25
D-dimer (220-500)	294	550	43,556	63,500
High-sensitivity troponin-T (hs-cTnT)	-	-	407	20,345

During the first part of the surgery, the hepatectomy, the patient was stable in a normal sinus rhythm, and no significant bleeding occurred during the first 60 minutes of surgery. The patient had short episodes of atrial fibrillation with a controlled ventricular response of around 60 bpm, with no further hemodynamic repercussions.

**Figure 3:**
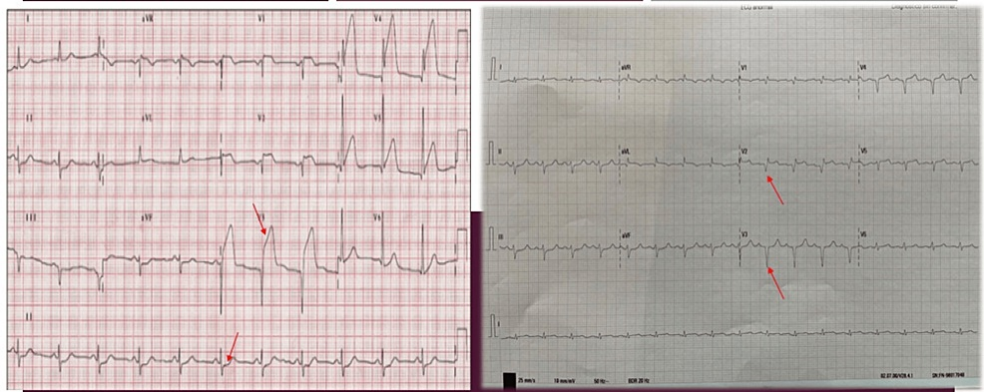
Electrocardiogram Left: Acute myocardial infarction electrocardiogram showing ST-segment elevation and decrease in derivation II. Right: Electrocardiogram after coronary thrombectomy. Q waves can be observed at this time in the affected territories.

However, a sudden onset event changed the course of the procedure. A left anterior fascicular block (LAFB) was noted alongside a drop in mean arterial pressure of 20 mmHg, and inferior derivation ST-segment elevation occurred a few seconds later (Figure 3).

This had no surgical correlation such as acute hemorrhage or vascular compression, as the hepatectomy had been completed and major veins were already ligated. Surgery was paused to stabilize the patient and hemodynamic instability became more profound. Norepinephrine and nitroglycerin were initiated as a treatment for the suspected myocardial infarction. After approximately two minutes, pulseless ventricular tachycardia ensued and cardiac defibrillation and advanced cardiac life support were initiated. The patient received 16 minutes of advanced cardiac life support, including 4 mg of epinephrine and three further cardiac defibrillations. Cardiac rhythm alternated between ventricular tachycardia and asystole. The return of spontaneous circulation was achieved with the support of epinephrine, norepinephrine, and isoproterenol perfusions and the resting rhythm was atrial fibrillation with an LAFB.

During the procedure, two further laboratory and thromboelastographic controls were performed (Table 1). These controls showed augmented fibrinolysis, high D-dimer levels, and increasing troponin levels. These levels of high-sensitivity troponin-T (hs-cTnT) ascended from 407 to 20,345 after 12 hours (Table 2). However, no significant coagulopathy was noticed, even after portal reperfusion. The liver graft transplantation was successfully performed without significant complications, utilizing the piggyback technique as is customary.

**Figure 4:**
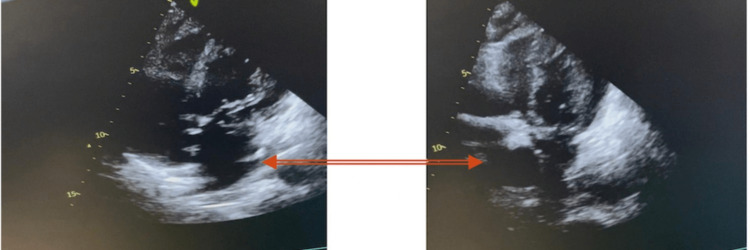
Post-surgical echocardiography Post-surgical images show a decrease in global contractility, apical-septal and apical-lateral wall akinesia/hypokinesia, and moderate mitral valve regurgitation. Images show the difference between diastole (left) and systole (right).

On arrival at the intensive care unit (ICU), the patient still had profound hemodynamic instability. Terlipressin was initiated at this point and the clinical situation improved despite moderate bleeding reported during the initial stage (600 ml/24 hours). Transthoracic echocardiographic controls were performed and compared with the pre-surgical study. It revealed a new-onset moderate to severe decrease in global contractility, apical-septal and apical-lateral wall akinesia/hypokinesia, and moderate mitral valve regurgitation (Figure 4).

Within 24 hours of his arrival at the ICU, the patient progressively improved, and the bleeding stopped, allowing his extubation and vasoactive medication to be titrated down. Coronary angiography showed diffuse atheromatosis and a sizeable thrombus over an ulcerated plaque in the middle third of the LMCA. A zotarolimus-coated stent was implanted in this location (Video 1).

**Video 1:** Coronary angiography

**Figure 5:**
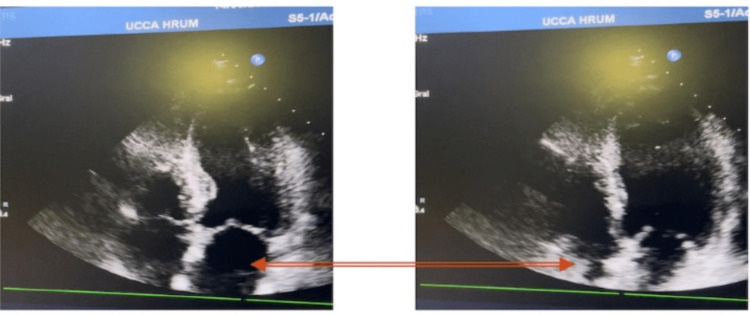
Post-myocardial reperfusion therapy echocardiography Post-myocardial reperfusion images (diastole (left) and systole (right)) show recovery of both left and right chambers. The ejection fraction was higher than 45%.

The patient was transferred to a surgical ward on postoperative day six and discharged home on postoperative day 21, experiencing no significant bleeding or thrombotic or cardiac events postoperatively. Further echocardiogram controls were performed, which showed progressive myocardial recovery (Figure 5). Both imaging and laboratory tests also showed the correct graft function. The medical records of the patient were examined 60 days after the operation. No instances of thrombotic complications were observed in imaging studies or clinic documentation, and the patient is presently not undergoing anticoagulation therapy.

## Discussion

Patients with liver disease face a higher susceptibility to ischemic strokes or venous thromboembolisms when affected by atrial fibrillation [5,6]. Emerging pharmacological treatments like DOACs are progressively being recognized for their superior pharmacological and clinical outcomes compared to vitamin K antagonists in addressing these conditions [7]. However, initial clinical trials of DOACs excluded patients with liver disease, and current drug guidelines advise against their use in cirrhotic individuals. Nevertheless, there is a growing body of evidence supporting the utilization of DOACs in advanced liver disease [7]. Recent studies examining the safety of DOACs in cirrhotic patients have produced mixed results, with some trials reporting favorable safety profiles [8]. In contrast, others have yielded inconclusive findings or indicated a potentially heightened risk of significant bleeding in individuals with more advanced liver disease [9]. Despite the increasing evidence suggesting the safety and efficacy of DOACs, including their unlikely association with significant liver injury regardless of existing liver impairment [10], there is limited literature available specifically addressing the use of dabigatran in this particular context.

The anticoagulant selection in our pretransplant ESLD patients is multifactorial. It is mainly dictated by patient factors such as the disease status, cardiologic affection, previous thrombus history, transplant eligibility, or monitoring of coagulation status. In this case, dabigatran was selected due to its more advantageous pharmacokinetic profile than other DOACs. This is especially true given the ability to rapidly reverse anticoagulation and restore hemostasis with idarucizumab before liver transplantation in an urgent manner.

Idarucizumab is an immunoglobulin fragment antigen-binding (Fab) reversal agent directed at dabigatran that was approved in 2015. In early trials, such as the RE-VERSE AD clinical trial, thrombin time (TT), activated partial thromboplastin time (aPTT), and ecarin time (ECT) were normalized in minutes following administration of the total 5 g dose. This is noteworthy as TT and ECT correlate with dabigatran concentration, and normalization of these laboratory values indicates low dabigatran levels, restoring the hemostasis [11]. To date, there are only two other published papers on effective dabigatran reversal with idarucizumab before liver transplantation [8,12]. However, there are some concerns regarding potential rebound thrombotic events after the use of idarucizumab, as shown by Rodrigues et al. [2]. This systematic review revealed an incidence of thrombotic events related to the use of this agent of 3.3% and an overall mortality rate of 9.5%.

In terms of the occurrence of thrombotic events associated with the need for an antidote, it seems that approximately 0.5% of patients undergoing surgery experience such events. The authors conclude that thrombotic events happen at a noteworthy frequency in a population with a significant short-term mortality risk. They emphasize the importance of prioritizing risk minimization strategies in this context and the need for solid evidence. Idarucizumab has a half-life of around 45 minutes, and all reported thrombotic events within 72 hours of its administration have been observed in patients who had not yet restarted anticoagulation [2,11,13,14]. Subsequent thrombotic events are more likely to be reflective of the underlying prothrombotic condition rather than a direct consequence of the reversal agent. Studies conducted on animals and healthy human volunteers have shown that idarucizumab does not possess any procoagulant properties. However, it is important to note that this evidence is not definitive and cannot determine whether the administration of idarucizumab may have contributed to myocardial infarction or not. In this case, a 5 g dose of idarucizumab was administered, as the surgery was assumed to be technically difficult due to the previous abdominal surgery. The precise effect of idarucizumab in this setting was difficult to predict as experience in our center was lacking and the literature review showed little evidence. Furthermore, it was difficult to predict, given the underlying coagulation pathophysiology of cirrhosis. We used the same dose that had been previously published in this clinical setting [12]. In conclusion, we could say that idarucizumab may not have a direct procoagulant effect, but in ESLD, there is a precarious balance of pro- and anti-coagulation [15], and therefore, correction of coagulopathy must be performed gradually and carefully and should be guided by clinical observations as well as test results.

Liver transplants involve many elements that may contribute to perioperative myocardial ischemia, including tissue trauma, general anesthesia, airway manipulation, fluid status, body temperature fluctuations, pain, bleeding, and preoperative fasting. The triggers above may result in perioperative inflammation, hypercoagulability, stress response, hemodynamic changes, hypothermia, hypoxia, and anemia, all playing potential prominent roles in the pathophysiology of acute myocardial ischemia. Perioperative myocardial infarction has a 30-day mortality of 11.4%, significantly higher than that in patients without myocardial injury [16].

VHA are point-of-care tests that allow a global assessment of coagulation using whole blood. The technology to enable this assessment has evolved, and, in our center, we use the QStat cartridge for Quantra. To monitor for hypercoagulability, albeit indirectly, we regularly assessed VHA values throughout the operation, together with a classical hemostatic test. To our knowledge, there is no report on how idarucizumab affects viscoelastic parameters in liver transplant settings. Moreover, there is scarce literature about OLT and its characteristic hemostatic changes assessed by this technology.

## Conclusions

This case report of an intraoperative myocardial infarction during liver transplantation after the reversal of dabigatran anticoagulation with idarucizumab could serve as an important lesson about the perils of reversing direct oral anticoagulants prior to OLT, especially given the underlying rebalanced coagulation system of cirrhosis, which can be prone to thrombosis. The use of DOACs in transplant recipients should be studied in much larger cohort studies, and both bleeding and thrombotic complications considered. Idarucizumab was shown to be an effective drug for reversing dabigatran anticoagulation for imminent surgery in our case. The procoagulant state induced by this reversal agent might be a potential perioperative trigger contributing to thrombotic events such as myocardial infarction.
